# Renoprotective effects of combined SGLT2 and ACE inhibitor therapy in diabetic Dahl S rats

**DOI:** 10.14814/phy2.12436

**Published:** 2015-07-14

**Authors:** Naoki Kojima, Jan M Williams, Tiffani N Slaughter, Sota Kato, Teisuke Takahashi, Noriyuki Miyata, Richard J Roman

**Affiliations:** 1 Department of Pharmacology and Toxicology, University of Mississippi Medical CenterJackson, Mississippi; 2 Pharmacology Laboratories, Taisho Pharmaceutical Co., Ltd.Saitama-shi, Saitama, Japan; 3 Pharmaceutical Business Planning, Taisho Pharmaceutical Co., Ltd.Toshima-ku, Tokyo, Japan

**Keywords:** ACE inhibitor, diabetic nephropathy, hypertension, insulin, SGLT2 inhibitor

## Abstract

This study examined whether control of hyperglycemia with a new SGLT2 inhibitor, luseogliflozin, given alone or in combination with lisinopril could prevent the development of renal injury in diabetic Dahl salt-sensitive (Dahl S) rats treated with streptozotocin (Dahl-STZ). Blood glucose levels increased from normoglycemic to hyperglycemic levels after treatment of STZ in Dahl S rats. Chronic treatment of Dahl-STZ rats with luseogliflozin (10 mg/kg/day) increased the fractional excretion of glucose and normalized blood glucose and HbA1c levels. Lisinopril (20 mg/kg/day) reduced blood pressure from 145 ± 9 to 120 ± 5 mmHg in Dahl-STZ rats, while luseogliflozin had no effect on blood pressure. Combination therapy reduced blood pressure more than that seen in the rats treated with luseogliflozin or lisinopril alone. Dahl-STZ rats exhibited hyperfiltration, mesangial matrix expansion, severe progressive proteinuria, focal glomerulosclerosis and interstitial fibrosis. Control of hyperglycemia with luseogliflozin reduced the degree of hyperfiltration and renal injury but had no effect on blood pressure or the development of proteinuria. Treatment with lisinopril reduced hyperfiltration, proteinuria and renal injury in Dahl-STZ rats. Combination therapy afforded greater renoprotection than administration of either drug alone. These results suggest that long-term control of hyperglycemia with luseogliflozin, especially in combination with lisinopril to lower blood pressure, attenuates the development of renal injury in this rat model of advanced diabetic nephropathy.

## Introduction

Diabetic nephropathy is the primary cause of end-stage renal disease worldwide. Despite the availability of new therapies that better control hyperglycemia and hypertension, the number of patients with CKD is still increasing (US Renal Data System, 2012). Effective control of blood glucose levels and blood pressure is important to prevent the progression of renal disease in diabetic patients. However, recent clinical data indicate that the current therapies to control hyperglycemia only slow, but do not prevent, the development of renal disease (Ohkubo et al. 1995; Ueda et al. 2003; Casas et al. 2005; Mann et al. 2008). Thus, there is a need to explore other therapies that might be more renoprotective.

The sodium glucose co-transporter 2 (SGLT2) is expressed in renal proximal tubule. Recently, several SGLT2 inhibitors have been developed that are effective in controlling blood glucose levels in patients with type 2 diabetes by increasing the excretion of glucose in the urine (Bailey et al. 2010; Wilding et al. 2013; Yale et al. 2013). However, it remains to be determined whether long-term control of hyperglycemia with these drugs will prevent the development of diabetic nephropathy.

The mechanism by which diabetes promotes renal injury also remains controversial. Several investigators have suggested that an increase in the filtered load coupled with upregulation of the expression of SGLT2 protein and enhanced glucose transport in the proximal tubule promotes glomerular hyperfiltration by reducing the delivered load of sodium to the Macula Densa and inhibiting tubuloglomerular feedback tone on the afferent arteriole (Af-art) (Noonan et al. 2001; Vallon and Thomson 2012). The subsequent increase in transmission of pressure to the glomerulus promotes mesangial matrix formation and the development of proteinuria and focal glomerulosclerosis. Several investigators have further suggested that blocking the increase in sodium and glucose co-transport in the proximal tubule of diabetic patients and animals would be renoprotective by chronically activating tubuloglomerular feedback (TGF) and to reduce hyperfiltration and glomerular capillary pressure (Vallon et al. 2013; Cherney et al. 2014; Schernthaner et al. 2014). On the other hand, it remains to be conclusively established that TGF plays important role in the long-term control of glomerular filtration rate (GFR) since previous studies have indicated that TGF responsiveness is rapidly diminished following elevations in salt intake or sustained elevations in tubular flow rate (Schnermann and Briggs 1990; Thomson et al. 1996, 1999). The results to date examining the renoprotective actions of SGLT2 inhibitors in clinical studies and various diabetic animal models are also not consistent. Our laboratory first reported that control of hyperglycemia with an SGLT2 inhibitor slowed but did not reverse the progression of glomerulosclerosis and renal interstitial fibrosis in 14-month-old type 2 diabetic rats with preexisting renal injury (Kojima et al. 2013). More recently, it was reported that control of plasma glucose levels with SGLT2 inhibitors reduced mesangial matrix expansion and albuminuria in type 2 db/db diabetic mice; however this model does not develop severe renal injury so it had no effect on plasma creatinine concentration or clearance (Nagata et al. 2013; Terami et al. 2014). The SGLT2 inhibitor, empagliflozin, was shown to reduce some of the early features of diabetic nephropathy including glomerular hypertrophy and glomerular matrix expansion in ob/ob type 2 diabetic animals. However, it has no effect on the glomerular hypertrophy or matrix expansion when hypertension was superimposed (Gembardt et al. 2014). A more recent clinical study has reported that 8-week treatment with empagliflozin reduced hyperfiltration in patients with type 1 diabetes but the study was not long enough to determine if it alters albuminuria or the later development of chronic kidney disease (Cherney et al. 2014). Others have reported that SGLT2 inhibitors reduced proteinuria in some clinical trials in type 2 diabetic patients, but it remains to be determined whether this is due to renoprotective effects in the glomerulus or secondary to the reduced GFR and the fall in the filtered load of protein in many of these studies (Schernthaner et al. 2014). Others have reported that knockout of the SGLT2 transporter in a streptozotocin (STZ)-induced diabetic mouse model does not prevent matrix expansion and renal interstitial fibrosis even though it controlled hyperglycemia and prevented hyperfiltration (Vallon et al. 2013). A more recent study in a type I diabetic Akita mouse model indicated that treatment with SGLT2 inhibitor attenuated the early features of diabetic nephropathy including hyperfiltration, renal hypertrophy and elevated urine albumin to creatinine concentration ratio (Vallon et al. 2014). However, it remains to be determined whether this treatment is effective in preventing the more advanced features diabetic nephropathy since this mouse model does not develop progressive proteinuria, matrix expansion and focal glomerulosclerosis, tubulointerstitital fibrosis or decrease in GFR (Breyer et al. 2005; Brosius et al. 2009; Kong et al. 2013). Recently, Slaughter et al. (2013) reported on a new type 1 diabetic nephropathy in STZ-treated diabetic Dahl salt-sensitive (Dahl S) rats that develops the early features of diabetic nephropathy including hyperfiltration, thickening of the basement membranes, mesangial matrix expansion and proteinuria and the more advanced features including progressive proteinuria, advanced matrix expansion with nodule formation, focal followed by global glomerulosclerosis, a fall in GFR and tubulointerstitial fibrosis. The mechanism for the increased sensitivity of the Dahl S rat to develop more severe renal injury and the advanced features of diabetic nephropathy remains to be determined but one likely factor may be that the myogenic and TGF response of the Af-art is impaired in Dahl S rats (Ge et al. 2014). Since it remains to be determined whether early intervention with an SGLT2 inhibitor to control hyperglycemia in an inducible type 1 model of diabetes can prevent the development of advanced diabetic nephropathy especially in conjunction with mild hypertension, the present study examined the effects of chronic administration of an SGLT2 inhibitor, luseogliflozin, given alone and in combination with ACE inhibitor on the development of renal disease following induction of diabetes in STZ-treated Dahl S (Dahl-STZ) rats.

## Methods

### General

Experiments were performed on 52, 8-week-old male Dahl S rats that were obtained from a colony maintained at the University of Mississippi Medical Center. The rats were housed in the Laboratory of Animal Facility at the University of Mississippi Medical Center, which is approved by the American Association for the Accreditation of Laboratory Animal Care. The rats were maintained on a standard rodent chow (Harlan Teklad 8640, Harlan Laboratories, Madison, WI) containing 1% NaCl and they had free access to food and water. All protocols were approved by the Animal Care Committee of the University of Mississippi Medical Center.

### Effect of a SGLT2 inhibitor on the development of renal injury in STZ-treated Dahl S rats

Eight-week-old Dahl S rats were fasted overnight. Diabetes was induced by a single injection of STZ (50 mg/kg, i.v.) (Sigma-Aldrich, St. Louis, MO). At the time of the injection, a 3.5 mm length of a long-acting Linplant insulin implant (Linshin Canada, ON, Canada) that delivers human recombinant insulin as a rate of ∼ 2 U/day/implant for up to 40 days was placed subcutaneously to prevent metabolic wasting and maintain blood glucose levels between 300 and 600 mg/dL. Three days after injection of STZ, blood glucose levels were measured using glucometer (Bayer HealthCare, Mishawaka, IN) to confirm that the rats were hyperglycemic in the expected range.

After the induction of diabetes, the rats were randomly assigned to five treatment groups. Group 1 (*n* = 9) served as the vehicle control group and was fed a Harlan Teklad 8640 powdered rat chow containing 1% NaCl and had free access to drinking water through the study. Group 2 (*n* = 9) received a selective SGLT2 inhibitor luseogliflozin (Kakinuma et al. 2010) that was synthesized by Medical Chemistry Laboratories in Taisho Pharmaceutical Co., Ltd. (Saitama, Japan) in the powdered rat chow at a concentration of 0.01% which delivered an average daily dose of 10 mg/kg. Group 3 (*n* = 9) received the ACE inhibitor, lisinopril, (Cayman Chemical, Ann Arbor, MI) in the drinking water to deliver an average daily dose of 20 mg/kg. Group 4 (*n* = 9) received luseogliflozin in the rat chow at an average daily intake of 10 mg/kg and lisinopril in the drinking water at an average daily intake of 20 mg/kg (combination therapy). Group 5 (*n* = 9) were implanted with a second 3.5 mm length of the Linplant insulin implant which was sufficient to restore blood glucose levels to normal in the STZ-treated Dahl S rats. The rats received another implant if the nonfasting blood glucose levels rose above 200 mg/dL at any time during the course of the study. A sixth group (*n* = 7) of untreated 9–10-week-old Dahl S rats were studied as a baseline control group for the assessment of renal hemodynamics and renal injury prior to the induction of diabetes. Blood samples were collected biweekly from the tail vein for measurement of blood glucose level and monthly for the measurements of glycosylated hemoglobin (HbA1c), plasma insulin and luseogliflozin concentrations. Twenty-four hour urine samples were collected via metabolic cages every 2 weeks to study changes in the excretion of glucose and proteinuria.

Total protein concentration was determined using the Bradford method (Bio-Rad Laboratories, Hercules, CA). Urinary glucose concentrations were determined using the glucose oxidase-peroxide method (Cayman Chemical). HbA1c levels were measured using a rapid HbA1c device (Bayer HealthCare, Sunnyvale, CA). Blood pressure was measured monthly using a tail-cuff device (Hatteras Instruments, Cary, NC). Plasma and urinary creatinine concentrations were determined using the Jaffe method (Wako Chemicals USA, Richmond, VA). Plasma insulin concentration was measured by ELISA kit (Mercodia, Uppsala, Sweden) and plasma luseogliflozin concentration was measured by LC/MS/MS. The fractional excretion of glucose was calculated as (urine glucose concentration / plasma glucose concentration) / (urine creatinine concentration / plasma creatinine concentration) × 100.

### Effects on renal hemodynamics

At the end of the chronic study, the rats were anesthetized with ketamine (20 mg/rat, i.m.) (Phoenix Pharmaceutical, St. Joseph, MO) and Inactin (50 mg/kg, i.p.) (Sigma-Aldrich, St. Louis, MO) and placed on a warming table to maintain body temperature at 37°C. The trachea was cannulated with PE-240 tubing to facilitate breathing and catheters were placed in the right femoral artery and vein for measurement of mean arterial pressure (MAP) and for i.v. infusions. The rats received an i.v. infusion of 0.9% NaCl solution containing 2% bovine serum albumin and 2 mg/mL FITC-labeled inulin at a rate of 6 mL/h for measurement of GFR. An ultrasound flow probe (Transonic System, Ithaca, NY) was placed on the renal artery to measure renal blood flow (RBF). After a 30-min equilibration period, urine and plasma samples were collected during an experimental period. Plasma and urine inulin concentrations were measured using a fluorescent microplate reader (BioTek Instruments, Winooski, VT) to calculate GFR. Filtration fraction was calculated as the ratio of GFR to renal plasma flow.

At the end of the renal clearance study, the left kidney of the rats was collected, weighed and fixed in 10% buffered formalin solution. Paraffin sections (3 *μ*m) were prepared and stained with Masson’s trichrome stain. Thirty glomeruli per section in the cortex were scored for the degree of glomerulosclerosis in a blinded fashion on a 0–4^+^ scale with 0 representing a normal glomerulus, 1^+^ representing loss of 1–25% of glomerular capillary area, 2^+^ representing a 26–50% loss, 3^+^ representing a 51–75% loss, and 4^+^ representing >75% loss of the capillaries in the glomerular tuft. The images were captured using a Nikon Eclipse 55i microscope equipped with a DS-Fil1 color camera (Nikon Instruments Inc., Melville, NY). The degree of renal interstitial fibrosis was assessed by measuring the percentage of blue staining of collagen in 10 adjacent cortical and medullary areas per slide using the NIS-Elements D 3.0 software (Nikon Instruments Inc.). The percentage of tubular necrosis in three adjacent regions of the medulla was determined by measuring the percentage of the field filled with protein casts that exhibit red fluorescence using the same software.

### Statistics

Mean values ± SE are presented. The significance of difference in mean values between and within groups was determined using an ANOVA followed by a Holm-Sidak test for preplanned comparisons using the SigmaPlot 11 software (Systat Software, San Jose, CA).

## Results

The daily intake of luseogliflozin averaged approximately 10 mg/kg in the rats treated with luseogliflozin alone or in the combination therapy group. Lisinopril intake averaged about 20 mg/kg in the lisinopril and combination therapy groups. Plasma concentration of luseogliflozin averaged approximately 30 ng/mL in the rats treated with luseogliflozin alone and in those that received combination therapy.

### Effects of the various treatments on food intake and body weight

**Figure 1:**
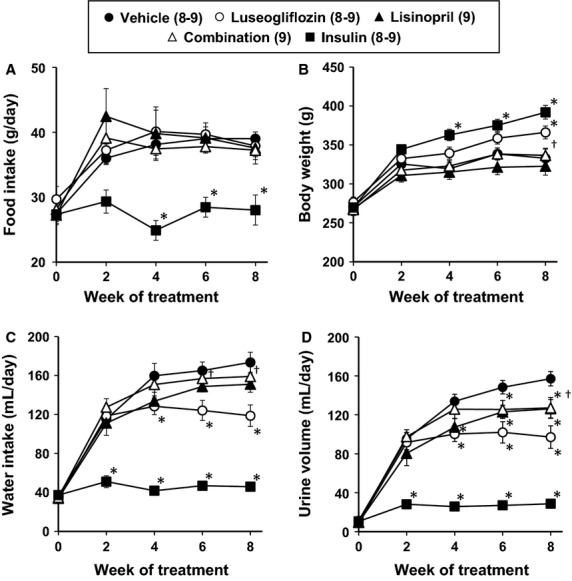
Effects of luseogliflozin, lisinopril, combination therapy and insulin on daily food intake (A), body weight (B), daily water intake (C) and daily urine volume (D) in Dahl-STZ rats. Numbers in parenthesis indicate the number of rats in which a complete data set was collected per group. *Indicates a significant difference from the corresponding value in vehicle-treated rats. ^†^Indicates a significant difference from the corresponding value in luseogliflozin-treated rats.

The results of these experiments are presented in Figure1A and B. Food intake increased after the induction of diabetes with STZ in all the groups except the rats treated with a high dose of insulin to maintain plasma glucose concentration in the normal range. Body weight remained relatively stable in the rats treated with vehicle or lisinopril. Body weight increased in rats treated with luseogliflozin compared to those treated with vehicle. Chronic treatment of the rats with insulin also increased body weight despite a decrease in food intake.

### Effects of the various treatments on water intake and urine flow

The results of these experiments are presented in Figure1C and D. Urine volume and water intake increased after the induction of diabetes in the vehicle-treated Dahl-STZ rats. Controlling blood glucose concentration with an insulin implant prevented the increase in water intake and urine volume. Water intake and urine volume initially increased to the same level as that seen in the vehicle control animals during the first 2 weeks of luseogliflozin treatment. Thereafter, water intake and urine volume were lower in the rats treated with luseogliflozin than in the control animals.

**Figure 2:**
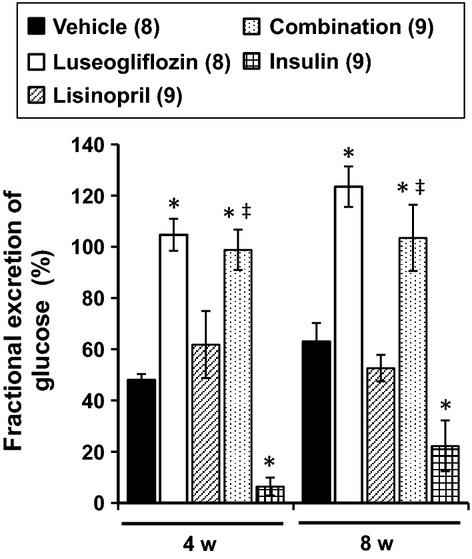
Effects of luseogliflozin, lisinopril, combination therapy and insulin on the fractional excretion of glucose in Dahl-STZ rats. Numbers in parenthesis indicate the number of rats in which a complete data set was collected per group. *Indicates a significant difference from the corresponding value in vehicle-treated rats. ^‡^Indicates a significant difference from the corresponding value in lisinopril-treated rats.

The effects of the various treatments on the fractional excretion of glucose are presented in Figure2. Luseogliflozin and combination treatment significantly increased the fractional excretion of glucose compared to that seen in the diabetic rats treated with vehicle. In contrast, lisinopril had no significant effect on the fractional excretion of glucose, while insulin markedly lowered glucose excretion relative to the level seen in the rats treated with vehicle.

### Effects of the various treatments on blood glucose levels

**Figure 3:**
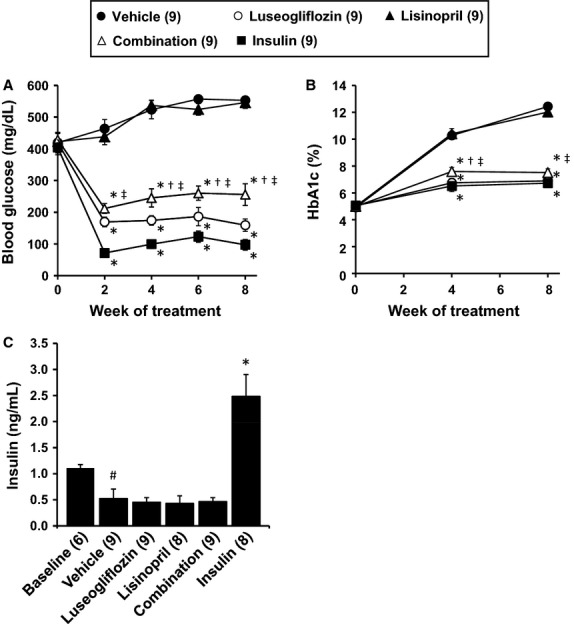
Effects of luseogliflozin, lisinopril, combination therapy and insulin on nonfasting blood glucose levels (A), HbA1c levels (B) and plasma insulin levels (C) in Dahl-STZ rats. Numbers in parenthesis indicate the number of rats in which a complete data set was collected per group. ^#^Indicates a significant difference from the corresponding values in the baseline control group of Dahl salt-sensitive rats. *Indicates a significant difference from the corresponding value in vehicle-treated rats. ^†^Indicates a significant difference from the corresponding value in luseogliflozin-treated rats. ^‡^Indicates a significant difference from the corresponding value in lisinopril-treated rats.

The effects of the various treatments on nonfasting blood glucose and HbA1c levels are presented in Figure3A and B. All of the Dahl-STZ rats were diabetic at the beginning of the study with blood glucose levels averaging approximately 400 mg/dL. Chronic treatment of the rats with luseogliflozin or insulin returned blood glucose levels and HbA1c levels to the normoglycemic range. Similar results were seen in the Dahl-STZ rats receiving combination therapy. In contrast, Dahl-STZ rats treated with lisinopril had no significant effect on blood glucose and HbA1c levels compared to the rats treated with vehicle.

### Effects of the various treatments on plasma insulin levels

The effects of the various treatments on plasma insulin levels are presented in Figure3C. Plasma insulin levels were significantly lower in Dahl S rats treated with STZ and a low-dose insulin implant to prevent wasting than the levels measured in 9–10-week-old nondiabetic control rats. Chronic treatment of the rats with a dose of insulin sufficient to normalize blood glucose levels raised plasma insulin levels about five-fold above the level measured in rats treated with vehicle. Neither, luseogliflozin or lisinopril had any effect on insulin levels relative to those seen in Dahl-STZ rats treated with vehicle.

### Effects of the various treatments on blood pressure and proteinuria

**Figure 4:**
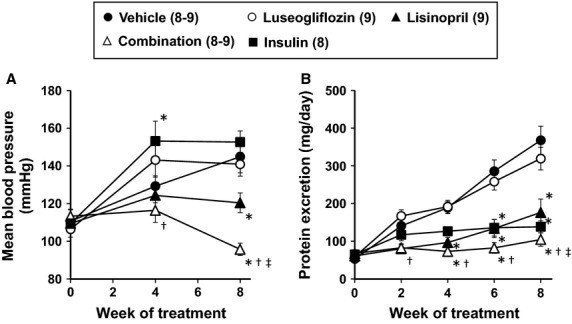
Effects of luseogliflozin, lisinopril, combination therapy and insulin on mean blood pressure (A) and urinary protein excretion (B) in Dahl-STZ rats. Numbers in parenthesis indicate the number of rats in which a complete data set was collected per group. *Indicates a significant difference from the corresponding value in vehicle-treated rats. ^†^Indicates a significant difference from the corresponding value in luseogliflozin-treated rats. ^‡^Indicates a significant difference from the corresponding value in lisinopril-treated rats.

The effects of the various treatments on mean blood pressure measured by tail cuff in the Dahl-STZ rats are presented in Figure4A. Blood pressure rose from 111 to 145 mmHg in Dahl-STZ rats treated with vehicle. Chronic treatment of the rats with luseogliflozin had no effect on mean blood pressure relative to the increase seen in rats treated with vehicle. In contrast, chronic treatment of the rats with lisinopril prevented the rise in blood pressure. Combination therapy reduced blood pressure to a greater extent than that seen in Dahl-STZ rats treated with luseogliflozin or lisinopril alone.

The effects of the various treatments on proteinuria are presented in Figure4B. Dahl-STZ rats exhibited a progressive increase in proteinuria throughout the study. Control of hyperglycemia with insulin prevented the development of proteinuria. In contrast, chronic treatment of the rats with luseogliflozin did not reduce proteinuria. Lisinopril significantly reduced proteinuria throughout the study and the combination therapy was more effective at preventing proteinuria than in rats treated with luseogliflozin or lisinopril alone.

### Effects of the various treatments on kidney weight

**Figure 5:**
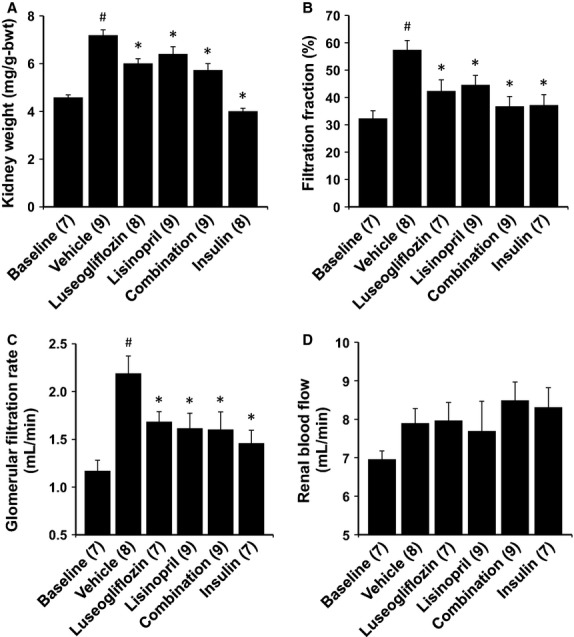
Effects of 2 months of chronic treatment with luseogliflozin, lisinopril, combination therapy and insulin on kidney weight (A), filtration fraction (B), glomerular filtration rate (C) and renal blood flow (D) in Dahl-STZ rats. Numbers in parenthesis indicate the number of rats in which a complete data set was collected per group. ^#^Indicates a significant difference from the corresponding values in the baseline control group of Dahl salt-sensitive rats. *Indicates a significant difference from the corresponding value in vehicle-treated rats.

The effects of the various treatments on kidney weight are presented in Figure5A. Kidney weight increased by 57% relative to the value seen in baseline control rats in Dahl-STZ rats treated with vehicle. Control of hyperglycemia with luseogliflozin or insulin significantly reduced the increase in kidney weight compared to that seen in rats treated with vehicle alone. Lisinopril and combination therapy also significantly reduced the degree of renal hypertrophy over the course of the study.

### Effects of the various treatments on renal hemodynamics

The effects of the various treatments on renal hemodynamics are presented in Figure5B, C and D. Dahl S rats treated with STZ exhibited significant hyperfiltration. GFR increased two-fold while RBF was unaltered relative to the levels measured in control Dahl S rats. Filtration fraction almost doubled. Control of hyperglycemia with luseogliflozin or insulin significantly reduced the degree of hyperfiltration compared to the rats treated with vehicle. Lisinopril also reduced GFR and the filtration fraction. Combination therapy reduced GFR and filtration fraction to the same extent as that seen in the rats treated with luseogliflozin or lisinopril alone.

### Renoprotective effects of the various treatments

**Figure 6:**
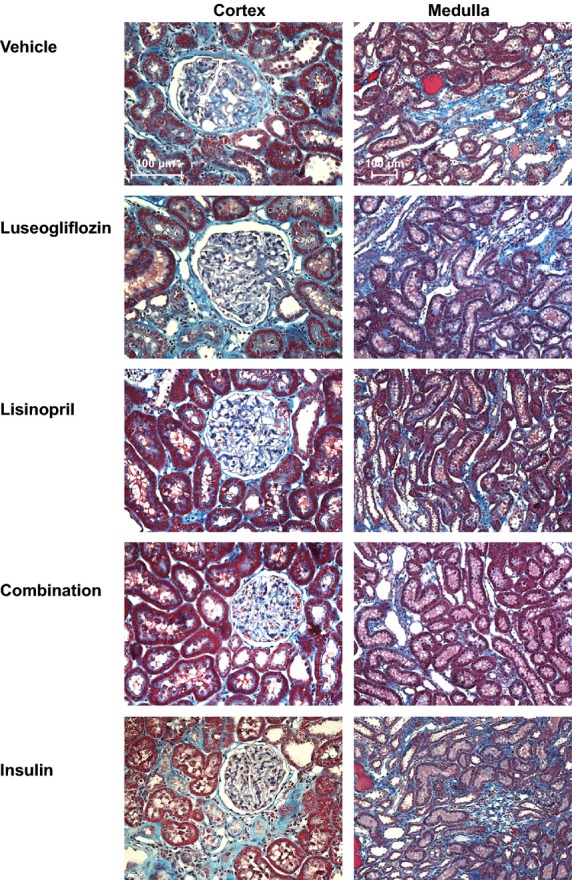
Representative appearance of the renal cortex (200×) and medulla (100×) stained with Masson’s trichrome in Dahl-STZ rats treated with vehicle, luseogliflozin, lisinopril, combination therapy and insulin.

**Figure 7:**
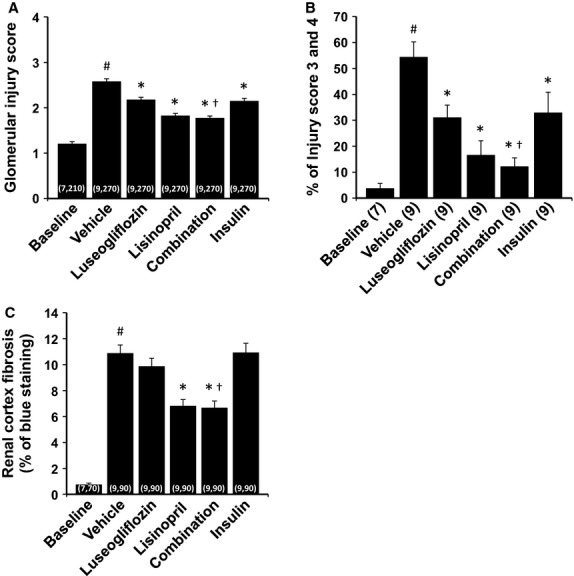
Effects of 2 months of chronic treatment with luseogliflozin, lisinopril, combination therapy and insulin on glomerular injury score (A), percentage of severe glomerulosclerosis (B) and renal cortical fibrosis (C) in Dahl-STZ rats. Numbers in parenthesis indicate the number of rats, glomeruli and areas in which a complete data set was collected per group. ^#^Indicates a significant difference from the corresponding values in the baseline control group of Dahl salt-sensitive rats. *Indicates a significant difference from the corresponding value in vehicle-treated rats. ^†^Indicates a significant difference from the corresponding value in luseogliflozin-treated rats.

**Figure 8:**
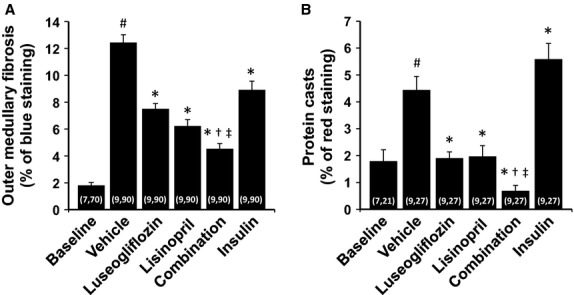
Effects of 2 months of chronic treatment with luseogliflozin, lisinopril, combination therapy and insulin on the degree of renal medullary fibrosis (A) and the formation of protein casts (B) in Dahl-STZ rats. Numbers in parenthesis indicate the number of rats and areas in which a complete data set was collected per group. ^#^Indicates a significant difference from the corresponding values in the baseline control group of Dahl salt-sensitive rats. *Indicates a significant difference from the corresponding value in vehicle-treated rats. ^†^Indicates a significant difference from the corresponding value in luseogliflozin-treated rats. ^‡^Indicates a significant difference from the corresponding value in lisinopril-treated rats.

The effects of the various treatments on the development of renal injury are presented in Figures6, 7 and 8. The kidneys of Dahl-STZ rats treated with vehicle exhibited severe thickening of the basement membranes, glomerular matrix expansion, focal glomerulosclerosis, renal interstitial fibrosis and tubular necrosis especially in the medulla. The mean glomerular injury score rose to 2.58 ± 0.06 indicative of loss of >25% of capillary area in Dahl-STZ rats treated with vehicle. More than half of the glomeruli scored had injury scores of 3 or 4 indicative of severe injury.

Chronic treatment of the rats with luseogliflozin significantly reduced the degree of glomerular injury, outer medullary fibrosis and protein casts which is an index of tubular necrosis. Chronic treatment of the rats with insulin also reduced the degree of glomerular injury and outer medullary fibrosis but it did not prevent the formation of protein casts in the medulla. Lisinopril reduced the degree of glomerular injury, renal fibrosis and the formation of protein casts. Combination therapy reduced outer medullary fibrosis and the formation of protein casts more effectively than that seen in rats treated with luseogliflozin or lisinopril alone.

## Discussion

Recently, several SGLT2 inhibitors that block glucose reabsorption in the proximal tubule via the sodium-dependent glucose transporter have been approved for the treatment of type 2 diabetes. Luseogliflozin is a relatively new member of this class with an IC_50_ value of 2.3 nmol/L for inhibition of SGLT2 (Yamamoto et al. 2011). It has been reported to effectively reduce hyperglycemia in both type 1 and 2 animal models of diabetes (Yamamoto et al. 2011). It was reported to slow the progression of renal injury in a rat model of type 2 diabetes with preexisting proteinuria and renal disease (Kojima et al. 2013). More recently, control of hyperglycemia with other SGLT2 inhibitors have been reported to prevent the early features of diabetic nephropathy including renal and glomerular hypertrophy, hyperfiltration and mesangial matrix expansion in mouse models of diabetic. However, it remains to be determined whether early and chronic control of hyperglycemia with luseogliflozin or other SGLT2 inhibitor will delay the development of the more advanced features of diabetic nephropathy including focal glomerular sclerosis and tubulointerstitial fibrosis in diabetic patients or an animal model of diabetes. Thus, the present study examined the effects of SGLT2 inhibition on the development of glomerulosclerosis, renal fibrosis and tubular necrosis in a Dahl-STZ rat model which has recently been reported to exhibit more of the features of advanced diabetic nephropathy (Slaughter et al. 2013).

Plasma concentration of luseogliflozin averaged 30 ng/mL in the rats treated with luseogliflozin. The unbound plasma concentration of luseogliflozin was calculated to be about 3.5 nmol/L (95% protein bound) which exceeds the IC_50_ value to block the SGLT2 transporter. Blood glucose and HbA1c levels were effectively controlled by luseogliflozin treatment alone or when given in combination with lisinopril in Dahl-STZ rats. The fall in blood glucose levels was associated with an increase in the fractional excretion of glucose to nearly 100% of the filtered load, indicating that the dose we gave was effective at blocking most of the reabsorption of glucose in the proximal tubule. Moreover, the hypoglycemic action of luseogliflozin was as effective as that seen in Dahl-STZ rats treated with insulin. The major differences were that urine flow and water intake increased markedly in STZ-treated Dahl S rats treated with luseogliflozin due to the loss of glucose in the urine, whereas water intake and urine flow did not increase in Dahl-STZ rats treated with insulin. Another difference is that plasma insulin levels were five times higher than control in the Dahl-STZ rats given insulin but plasma insulin levels were low in Dahl-STZ rats treated with luseogliflozin.

Dahl S rats treated with STZ indicated by a 57% increase in kidney weight (renal hypertrophy), hyperfiltration as indicated by a doubling of GFR and a rise in filtration fraction. Control of hyperglycemia with luseogliflozin significantly blunted the increase in GFR and the degree of renal hypertrophy. Previous investigators have reported that the elevation in GFR in diabetic animals is associated with upregulation of SGLT2 coupled sodium reabsorption in the proximal tubule which promotes a TGF mediated dilation of the afferent arteriole secondary to a fall in sodium delivery to the Macula Densa (Noonan et al. 2001; Vallon and Thomson 2012). The present findings are consistent with this view and indicate that inhibition of SGLT2 with luseogliflozin may reduce hyperfiltration in diabetic animals by increasing sodium delivery to the Macula Densa and activating TGF (Thomson et al. 2012). On the other hand, sustained elevations in TGF decrease the sensitivity of this feedback mechanism, so it is also possible that the fall in GFR in rats treated with luseogliflozin which might activate TGF and insulin that cannot, simply reflects a reduction in renal hypertrophy and glomerular size secondary control of hyperglycemia. This conclusion also seems more likely than the TGF hypothesis since both myogenic and TGF responsiveness are markedly impaired in Dahl S rats (Ge et al. 2014) so it is unlikely that the prevention of hyperfiltration could be due to activation of TGF.

Chronic treatment of the Dahl-STZ rats with luseogliflozin also reduced the degree of glomerular injury, outer medullary fibrosis and the formation of protein casts which is an index of tubular necrosis. However, it did not reduce proteinuria despite controlling blood glucose concentration. The mechanism by which luseogliflozin reduces glomerular injury remains to be determined. One possibility is that it blunts hyperfiltration and the rise in glomerular capillary pressure that stimulates mesangial matrix expansion, loss of podocytes and damage to the glomerular permeability barrier. It also could reduce renal inflammation and the release of cytokines and growth factors by controlling hyperglycemia. On the other hand, the failure of luseogliflozin to prevent the development of proteinuria despite reducing glomerular injury was unexpected. We suspect that this may be due to the chronic inhibition of sodium and glucose transport in the proximal tubule which may reduce the reuptake of filtered protein secondary to increased flow rate and reduced contact time in the proximal tubule.

Chronic treatment of the Dahl-STZ rats with insulin completely prevented renal hypertrophy and significantly reduced the degree of renal hyperfiltration and glomerular injury in Dahl-STZ rats. It also markedly attenuated the development of proteinuria. This appears to be mostly due to the prevention of hyperglycemia that promotes the increase in GFR. However, it was surprising that control of hyperglycemia with insulin did not attenuate the development of renal cortical fibrosis or the formation of tubular casts which is an index of tubular necrosis. The failure of insulin to reduce tubular necrosis suggests that the renal injury might be secondary to the profibrotic and growth promoting effects of the elevated circulating levels of insulin in this diabetic model. Indeed, our finding that control of hyperglycemia with luseogliflozin which did not increase plasma insulin levels, did reduce the degree of tubular necrosis is consistent with this view. On the other hand, insulin also promotes sodium retention and increased blood pressure to a greater extent than that seen in Dahl-STZ rats treated with vehicle. The greater rise in blood pressure in this animal model that lacks RBF autoregulation (Ge et al. 2014) may have increased glomerular injury even in the absence of hyperglycemia.

We also compared the degree of renal protection afforded by luseogliflozin with that seen in rats treated with an ACE inhibitor, which is the standard of care in diabetic nephropathy. Long-term blockade of the renin angiotensin system with lisinopril reduced blood pressure in the Dahl-STZ rats. Lisinopril reduced proteinuria and the degree of renal hypertrophy and hyperfiltration relative to vehicle-treated animals. It also reduced the degree of glomerular injury, renal fibrosis and tubular necrosis. The effect of lisinopril on proteinuria is likely due to the fall in blood pressure and glomerular capillary pressure secondary to dilation of the efferent arteriole following blockade of the renin angiotensin system. Indeed, we found that filtration fraction, GFR and the filtered load of protein were all reduced in rats treated with lisinopril. Blockade of the renin angiotensin system also reduces oxidative stress and the stimulatory effects of angiotensin II on the expression of a various growth factors and cytokines known to contribute to renal injury.

Blood glucose and HbA1c levels were effectively controlled in the rats that received combination therapy with luseogliflozin and lisinopril. Blood pressure was significantly reduced in the rats that received combination therapy more than that seen in animals treated with either agent alone. The greater fall in blood pressure may reflect the combined effect of luseogliflozin to reduce plasma volume acting synergistically with blockade of the effects of the renin angiotensin system with lisinopril on vascular tone. Moreover, the degree of proteinuria was lower in the rats receiving combination therapy than that seen in the animals treated with luseogliflozin or lisinopril alone. This probably is due to the effective dual control of both hyperglycemia and blood pressure in the animals receiving combination therapy.

### Perspectives

The present results indicate that chronic administration of a selective SGLT2 inhibitor luseogliflozin is very effective in controlling hyperglycemia in Dahl-STZ model of diabetic nephropathy. It reduced renal hyperfiltration and glomerular and tubular injury but not the development of proteinuria. Lisinopril reduced the development of proteinuria, renal hyperfiltration, glomerular injury and renal fibrosis due to a reduction in blood pressure in this model. Combination therapy with luseogliflozin and lisinopril was more effective at lowering blood pressure and preventing the development of proteinuria and renal injury than administration of either agent alone. These findings are consistent with our recent findings that administration of luseogliflozin alone and in combination with lisinopril slowed the progression of renal disease in a type 2 model of diabetic nephropathy with preexisting renal injury (Kojima et al. 2013). The results suggest that effective control of both hyperglycemia and blood pressure with combined treatment with SGLT2 and ACE inhibitors is an attractive therapeutic option for treatment of both type 1 and 2 diabetes that can slow the development and progression of CKD.
